# The influence of body orientation on length judgements

**DOI:** 10.1177/03010066241308139

**Published:** 2025-01-09

**Authors:** Federica Scarpellini, Jeroen B. J. Smeets

**Affiliations:** 1190 Vrije Universiteit Amsterdam, The Netherlands; 1190 Vrije Universiteit Amsterdam, The Netherlands

**Keywords:** proprioception, retinal size, multisensory perception, body perception

## Abstract

Perceiving the size of a visual object requires the combination of various sources of visual information. A recent paper by Kim et al. (Body Orientation Affects the Perceived Size of Objects. Perception 2022, 51: 25–36) concluded that body orientation played a substantial role. The present paper aims to answer the question of whether the reported effect of body orientation on visuo-haptic size matching was due to effects on the visual or the haptic judgements of size. To do so, we used a within-participant design combining an experiment using visuo-haptic size matching with two experiments that assessed the visual and haptic size-percept using free magnitude estimation. Our experiments produced a systematic visuo-haptic mismatch, but the sign of the mismatch was opposite to that of the original study. Moreover, our study did not reveal a systematic effect of body orientation on this mismatch. Thirdly, we found that the mismatch we determined from participants matching a visual and haptic percept was considerably smaller than the mismatch we derived from their visual and haptic size estimates. In summary, our results emphasise that conclusions about the perceived size of objects are very sensitive to details of the experimental approach.

Judging the one-dimensional size of an object (e.g., its length) one is looking at seems a rather straightforward task. Of course, this percept can be influenced by various visual illusions such as the Müller-Lyer illusion (de Brouwer et al., 2014; Stuart et al., 1984), the Ebbinghaus illusion (Pavani et al., 1999; Smeets et al., 2020), the Ponzo illusion (Brenner & Smeets, 1996; Gillam, 1973), the horizontal–vertical illusion (Avery & Day, 1969; Vishton et al., 1999), or the moon illusion (Boring, 1943). Such illusions capitalise on the fact that judging size involves the combination of various information sources or cues that all may be biased: illusions induce one or more of such biases. This combination of cues generally relies on assumptions or knowledge, for instance when viewing familiar objects (Predebon, 1992; Smeets et al., 2022). As the way one combines these sources differs between individuals, the effectiveness of visual illusions varies between individuals (de Grave et al., 2004). Most remarkably, this difference in sensitivity is specific to each type of illusion such that the susceptibility to one illusion is only very weakly correlated with the susceptibility to other illusions (Axelrod et al., 2017; Cretenoud et al., 2019; Grzeczkowski et al., 2017).

There are various ways to determine the perceived size of an object. A frequently used technique is a matching technique: participants can manipulate the size of one object (e.g., by using the keyboard or mouse) to match the size of the other object. If only one object is in an illusory context and the other is not, the difference in set size is regarded as the result of the illusion. One assumes generally that in such a matching task, it is irrelevant which of the two objects is adjustable. However, this is not the case for all matching tasks. For example, matching your grip aperture to the size of a visual stimulus does not correspond to adjusting the size of the stimulus to match your grip aperture (Kopiske & Domini, 2018). Similar inconsistencies have been reported for position matching (Kuling et al., 2017). Such inconsistencies are idiosyncratic: they are reproducible within participants but vary between participants (Kuling et al., 2016, 2017).

A possible explanation for the idiosyncrasies in the inconsistencies and the susceptibility to illusions is that there are many ways to judge size. To determine the size of an object from viewing it, one could interpret the size of the retinal image using an estimate of distance. There are abundant distance cues (Brenner & Smeets, 2018), and the preference for using a cue (or a certain combination of cues) could depend on the task and personal preferences. Alternatively, one could use the amplitude of saccades across the object combined with retinal error instead of the retinal size, again scaled for distance with one or more distance cues. It might be that each individual uses a different strategy and thus might be differently biased. It might even be that participants do not perform any distance scaling and report the angular size (in degrees) rather than the linear size (in cm), as has been discussed to explain the moon illusion (Plug & Ross, 1994). Size illusions such as the moon illusion are thus based on a combination of various mechanisms of size judgement (Coren & Aks, 1990). Such a combination of mechanisms, all with specific biases, has as a side effect that a specific judgement of size can easily be inconsistent with other judgements related to that size (Smeets & Brenner, 2023).

One of the mechanisms underlying the moon illusion depends on posture: the illusion changes if viewed with the head upside down (Coren, 1992). Other visual size judgements have also been reported to depend on body posture (Kim et al., 2022). This is remarkable, as most theories about visual perception regard visual perception of a static scene as mere processing of visual information. Why would the orientation of body parts other than the eye affect visual perception? To answer this question of why such irrelevant sensory modalities might affect visual perception, we set out to find out what the cause of these effects is.

A change in posture can be sensed in many ways, for instance, by using signals from the retina, otoliths, muscle spindles, and graviceptors in the trunk (Mittelstaedt, 1998). Exploring the various combinations of these information sources would result in an enormous experiment. Therefore, we decided to keep visual and vestibular information constant and restrict ourselves to a limited set of sources of somatosensory information (including proprioception). We did so by keeping the head in the same orientation (oriented 45° upwards). We furthermore only used two orientations of the body (standing and lying), with the lower arms pointing in the sagittal direction. These two postures differ in the orientation of the body relative to the head and relative to gravity. The latter influences the activity of the muscles around the elbow to stabilise the lower arm and may therefore influence haptic perception of length (Smith et al., 2009; Supralk et al., 2007; but see Kuling et al., 2013). To investigate the potential contribution of this effect directly, we added a third posture: lying with a different orientation of the lower arm. Despite these restrictions, two issues remain.

The first issue is the task one should use to assess the percept. One could use a comparison task (Kim et al., 2022): asking the participants to compare the size of a visual object with a reference object one is holding (i.e., haptic size perception). This can be done using a staircase procedure two-alternative forced-choice paradigm (Kim et al., 2022), or by asking participants to manipulate the visual object until its size matches that of the reference object. Such comparison methods have a serious disadvantage: if one finds an effect of posture on this task, one is not sure whether it is the perception of the visual object that is affected or the perception of the haptic reference. We therefore decided to additionally use magnitude estimation (Moskowitz, 1977) to obtain independent measures of the visual and haptic percept. We used both approaches (matching and magnitude estimation) to study the effects of posture on the judged size of the reference object. This made it possible to check whether the biases between vision and haptics were consistent for the two approaches.

A second issue is how to determine the presence of idiosyncratic effects of posture, that is, that a certain change of posture has reproducible effects on posture that differ between participants. We will measure each posture in a separate block. How do we know that idiosyncratic differences in estimates between two blocks are due to the associated change in posture, and not due to random variations over time? We have no perfect solution to this question but can estimate a lower limit for the idiosyncratic effect. For this we capitalised on a side effect of our design: we have two postures that only differ in the orientation of the lower arm. We use the variability in size judgement between these two postures as our estimate for random variations. We regard the additional variation in judgements caused by a larger change of posture as an indication of the idiosyncratic effects of posture change on size perception.

The present study thus aims to answer three questions:

1. Do changes in posture have systematic effects on visual and haptic size judgements?
2. Do changes in posture have idiosyncratic effects on visual and haptic size judgements?
3. Are the differences between visual and haptic size estimations (and the possible effect of posture on them) consistent with biases in visuo-haptic matching?

## Methods

### Design

**Figure 1.**
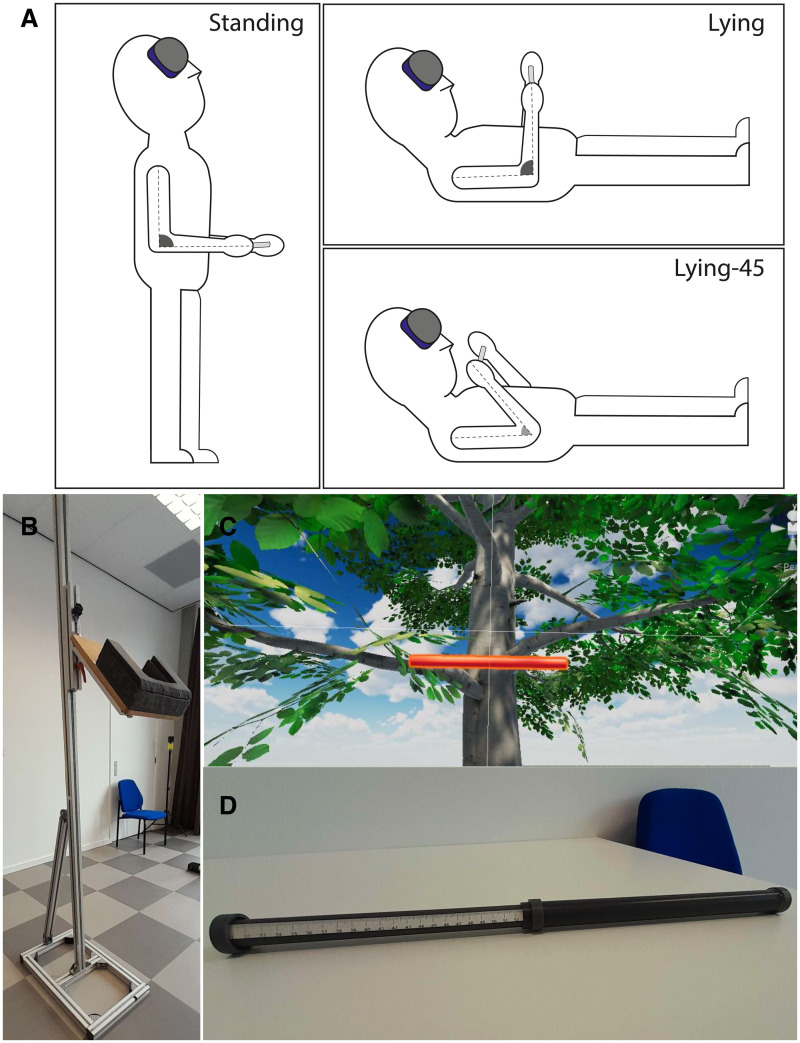
Set-up and stimuli. (A) The three postures used in the experiment. (B) The support helped participants to keep the head in a 45° upward looking orientation. (C) Part of the visual stimulus, including the bar in front of a tree. (D) The bar subjects held in their hand in the visuo-haptic matching and haptic estimation tasks.

We compared size perception in three postures (Figure 1A). In all postures, the participants had their head tilted looking upwards at about 45°. In addition to the *standing* posture (with the upper arms parallel to the trunk and a 90° elbow angle), we used two postures in which the participants were lying that only differed in elbow angle. In the *lying* posture, the elbow angle was 90°, just as in the standing posture. In the lying-45 posture, the elbow was more flexed: 45°. In all postures, we presented for each participant five different bar lengths to make sure that participants were judging the length of the bar reliably. The set of bar lengths was tailored for each participant. One bar length corresponded with the shoulder width of the participant ( $l_{0}$ ); the other four were 3 cm and 6 cm longer or shorter.

Each participant performed three tasks: *visual estimation*, *haptic estimation*, and *visuo-haptic matching*. In the first two tasks, participants were viewing or holding a bar (details in the section ‘Set-up and Stimuli’). They judged the perceived length by magnitude estimation: verbally reporting a number that was proportional to the lengths using a self-selected unit of measurement. In the visuo-haptic matching task, participants viewed a bar (presented by means of a virtual reality [VR] headset) which length was set by the experimenter. At each trial, the participants received a haptic bar at a random initial length. They were asked to lengthen or shorten the haptic bar until they considered its length matching that of the visual bar.

### Set-up and Stimuli

We aimed to have the three postures only differ in somatosensory information. To keep the head in the same (45° upward) orientation, we constructed a head support that was adjustable in height. For lying, it was combined with a physiotherapy bed on which the participant could lie down. For standing (Figure 1B), it was adjusted in height to match individual height. We furthermore created a construction to indicate the desired elbow angle in the two lying conditions.

To present a visual bar in a way that is independent of body posture, we used a HTC Vive Pro Eye. This provides a resolution of 1,440 × 1,600 pixels per eye at about 13 pixels/° for each eye. The refresh rate is 90 Hz. The VR environment was built in the Unity game engine 2019.4 LTS. The constructed virtual environment reproduces a natural environment with a tree at about 3 m distance and clouds in the background (Figure 1C). The participant wore the headset and viewed this environment in all three tasks. These items provided participants with a visual scale to judge the length of a horizontal bar (a simulation of a red 3 cm diameter cylinder) that was positioned at approximately 2.5 m from the participant in the visual estimation and visuo-haptic matching tasks. This distance is close to the specific distance one assumes by lack of accurate distance information (Gogel, 1969; Gogel & Tietz, 1973). We aim to study size perception by judging the length of a bar. One could perform this task also without perceiving size, but by keeping track of the positions of the endpoints relative to visual features across trials. To prevent participants from doing so, we vary the position of the bar from trial to trial (randomly chosen from a 3 × 3 grid of points extending 6 × 6 cm. Moreover, by presenting the visual bar at a different distance than the haptic bar, we ensured that in the matching task, participants did not match perceived endpoints or angular extent, but their visual and haptic percept of object size.

The haptic bar (Figure 1D) is composed of two hollow lightweight cylinders made of a light material. On one end of each cylinder, there is a 3 cm diameter handle. At the other end, the smaller of the two cylinders can slide within the larger one. In this way, either the experimenter or the participant can vary the total length of the bar. In the haptic estimation task, the experimenter moves the cylinders until the length of the bar (visible on a ruler attached to the inner bar) matches the desired length of that trial. Subsequently, the bar is fixed at that length through a screw, and handed to the participant. In the visuo-haptic matching task, the participant moves the two cylinders until the bar feels equally long as the visual bar. The experimenter can read the set length of the bar on the ruler.

### Participants

**Figure 2.**
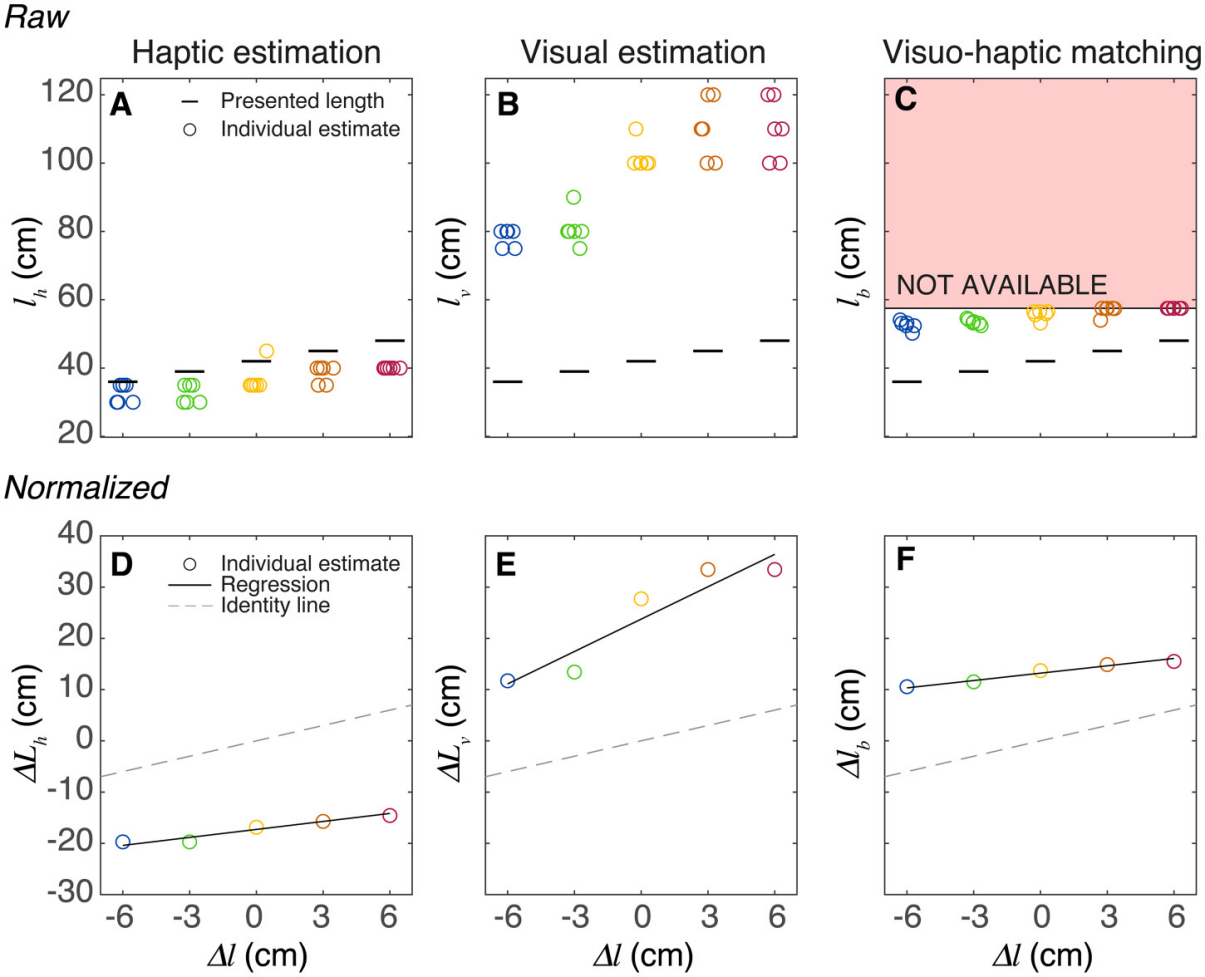
Data analysis for a single participant. (A–C) Raw data for the three tasks while standing. For the five bar lengths, the results of all six trials of this participant are indicated by circles. (C) The set length of the haptic bar ( $l_{b}$ ) as a function of the variation of the length of the presented visual bar. When trying to match the longest visual bar, this participant reached the limits of the length range in total in 39 instants, so we label the matching data for this participant as unreliable in the figures with individual data (see the section ‘Discussion’). D–E: The data of panels A and B after normalising the estimates. F: The data of panel C after subtracting the real length. In panels D–F, the circles indicate the average performance of this participant, and the dashed line indicates performance that is identical to the presented lengths.

The Scientific and Ethical Review Board of the Faculty of Behavioural and Movement Sciences approved this study (VCWE-2023-091). Participants were informed about the experiment and provided written consent before participating. For this explorative study, we have no good estimate of the within and between participant variability. We therefore based our number of participants on the numbers used in other studies (Kim et al., 2022; van der Hoort & Ehrsson, 2016; Winter et al., 2005). We planned to include 25 participants, one more than used in all the studies mentioned above. During the measurements, we observed that some participants for some bar lengths reached the upper limit of possible bar lengths when matching the hand-held bar to the visual bar (Figure 2C). We then decided to recruit new participants who performed the conditions in the same order as the participants who reached the limit. This would allow us to prevent any influence of this ceiling effect. After having recruited seven new participants, we realised that the participants who reached the limit in the matching task were unaffected by this limit in their visual and haptic estimates. We therefore decided that it would be better to include all participants (in total 32) to prevent any selection bias by excluding participants with the largest visuo-haptic mismatch. To clarify where potential ceiling effects may play a role, we indicate in the figure with individual matching data which participants were affected by this limitation (see the section ‘Data Analysis’). The participants’ shoulder widths were 40.5 ± 2.9 cm (μ ± σ).

### Procedure

The experiment consisted of three blocks; in each block, the participants performed the three tasks in a single posture. Each block started with the two estimation tasks (each participant performed these in a random order that was the same for the three postures), followed by the matching task. We presented the matching task as the last one to ensure that participants could familiarise themselves with haptic and visual estimating before performing the matching task. We reasoned that if participants had performed the matching task before an estimation, they might transform that estimation task to a task in the other modality. When presenting a scene in VR, we switched off the head-tracking when performing the task to ensure a constant visual field. Before transitioning to a new posture, we switched the head-tracking on allowing exploration of the scene. For each combination of posture and task, we presented the five lengths six times, in random order. Participants could take a break at each posture change. The total experiment lasted less than 2 hr.

### Data Analysis

All data analysis was performed using custom-build scripts using MATLAB. We started by identifying the participants whose matching results might be affected by reaching the upper limit of the haptic bar. We reasoned that given the range of presented lengths and the variability in responses, a few times reaching the limits won’t affect the parameters of the fitted line. We, therefore, decided to use 15 trials as the limit: we will regard the matching results of participants that reached the limit in more than 15 trials as questionable. Participants 5, 7, 9, 15, 16, 19, 24, 25, 29, and 31 were affected and will be labelled as unreliable in the figures with individual data.

We used free magnitude estimation to assess the perceived lengths of the various bars. In these haptic and visual tasks, we expect participants to express the length of the bars proportional to the actual length, using a scaling factor ( $s_{h}$ and $s_{v}$ ) that is unknown to the experimenter. The average length presented in both tasks is the shoulder width $l_{0}$ , and the presented length in a trial is $l_{0}+\Delta l$ (Δl∈{−6,−3,0,+3,+6}) .

(1) $$l_{h}=s_{h}(l_{0}+\Delta l)$$

(2) $$l_{v}=s_{v}(l_{0}+\Delta l)$$

To make the results of the participants better comparable (i.e., correct for individual misjudgements of what a cm is), we normalised the length estimates (yielding $L_{h}$ and $L_{v}$ ). We did so by multiplying all estimates by the ratio between the actual average length ( $l_{0}$ ) and the average estimate across bar lengths and tasks $\left(\frac{\left(s_{h}+s_{v}\right)}{2}l_{0}\right)$ .

(3) $$L_{h}=\frac{2s_{h}(l_{0}+\Delta l)}{\left(s_{h}+s_{v}\right)}$$

(4) $$L_{v}=\frac{2s_{v}(l_{0}+\Delta l)}{\left(s_{h}+s_{v}\right)}$$

To have a measure that is independent of the subjects’ shoulder width, we subtract the average length, resulting in the *normalised deviations* $\Delta L_{h}$ and $\Delta L_{v}$ :

(5) $$\Delta L_{h}=\frac{2s_{h}(l_{0}+\Delta l)}{\left(s_{h}+s_{v}\right)}-l_{0}=\frac{\left(s_{h}-s_{v}\right)}{\left(s_{h}+s_{v}\right)}l_{0}+\frac{2s_{h}}{\left(s_{h}+s_{v}\right)}\Delta l$$

(6) $$\Delta L_{v}=\frac{2s_{v}(l_{0}+\Delta l)}{\left(s_{h}+s_{v}\right)}-l_{0}=\frac{\left(s_{v}-s_{h}\right)}{\left(s_{h}+s_{v}\right)}l_{0}+\frac{2s_{v}}{\left(s_{h}+s_{v}\right)}\Delta l$$

We will fit a linear regression to the haptic and visual normalised deviations as a function of bar length variation ( Δl ) for each participant in each posture (Figure 2).

(7) $$\Delta L_{h}=H_{0}+H\;\Delta l$$

(8) $$\Delta L_{v}=V_{0}+V\;\Delta l$$

Combining equations (5)–(8), one can derive that the measured ratio of the visual and haptic slopes V/H will correspond to the ratio of the scaling factors $s_{v}/s_{h}$ , and is thus independent of $l_{0}$ . In the next paragraph, we will use this ratio to predict how participants will perform in the matching task.

Based on the above equations, we can predict what will happen when participants are asked to match the haptic bar length to the length of a visual bar, that is, how they would perform the visuo-haptic matching task. In this task, the length of the visual bar is varied in the experiment; its length will be $l_{0}+\Delta l$ , and this length will according to equation (2) be perceived as $s_{v}(l_{0}+\Delta l)$ . The participant varies the bar length ( $l_{0}+\Delta l_{b}$ ) in such a way that the haptic length judgement of equation (1) equals the visual one $\left(l_{h}=l_{v}\right)$ :

(9) $$s_{h}(l_{0}+\Delta l_{b})=s_{v}(l_{0}+\Delta l)$$

Based on equation (9), we can predict which haptic bar length participants will set for each visual bar length:

(10) $$\Delta l_{b}=\left(\frac{s_{v}}{s_{h}}-1\right)l_{0}+\frac{s_{v}}{s_{h}}\Delta l$$

Given the fact that the ratio of the scaling factors $s_{v}/s_{h}$ equals the measured ratio of the visual and haptic slopes V/H we can write:

(11) $$\Delta l_{b}=\left(\frac{V}{H}-1\right)l_{0}+\frac{V}{H}\Delta l$$

Equation (11) thus provides a prediction for how a participant will perform in the visuo-haptic matching task based on the ratio of the slopes obtained from the two estimation tasks. We will compare the consistency between estimation and matching by comparing this prediction based on estimation with the participant's performance in the matching task. To analyse the data of the matching experiment, we will therefore subtract $l_{0}$ from the matched bar length to obtain the matched deviation $\Delta l_{b}$ .

### Statistical Tests

The primary goal of the paper is to explore whether there are any systematic differences between postures in estimating or matching visual and haptic lengths. For this, we provide plots of the individual data and will report means, standard deviations and 95% confidence intervals for various variables. Reporting 95% confidence intervals is related to tests like the *t*-test. If such a confidence interval includes zero, we can safely claim that the data do not support a difference. Finding a confidence interval that does not include zero, on the other hand, does not imply a meaningful effect. For this, we should have formulated our hypothesis (and which variable to look at) before looking at the data. As we did not, we would formulate a hypothesis post hoc, which would be ‘Hypothesizing After the Results are Known’ (HARKing; Kerr, 1998), a questionable research practice. Therefore, we do not use statistical null hypothesis testing (Brenner, 2016; Simmons et al., 2011).

In addition to our exploration of the effects of posture on a group level, we are also interested in individual differences. If posture has an idiosyncratic effect on size judgements, we expect to find a larger difference when the whole body changes orientation, than when only the lower arm changes orientation. As the differences might be in different directions for different participants, we will focus on the variance of the effect of orientation change. We will use Levene's test for equality of variances to test whether the between-participant variability in the effect of changing whole-body orientation is larger than the variability in the effect of changing elbow angle. If so, we would have clear evidence for idiosyncratic effects of posture on size judgements.

## Results

**Figure 3.**
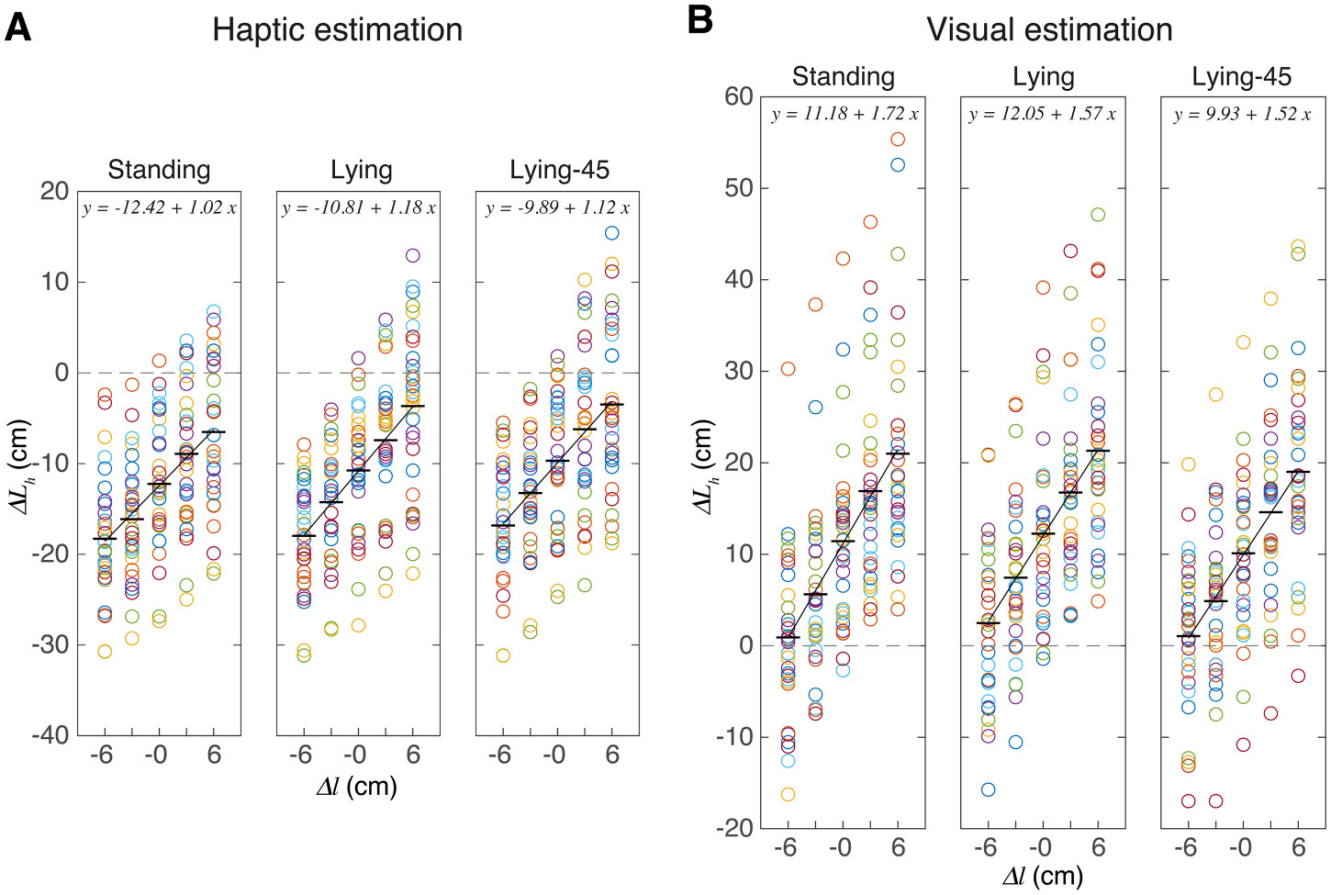
Individual data of the haptic and visual estimation tasks for the three postures. Symbols represent the average normalised deviation of a participant for a certain bar length. Horizontal lines indicate the mean across participants, and the oblique lines the linear fit. Note that on average, the visual bars were judged about 20 cm longer than haptic bars.

**Figure 4.**
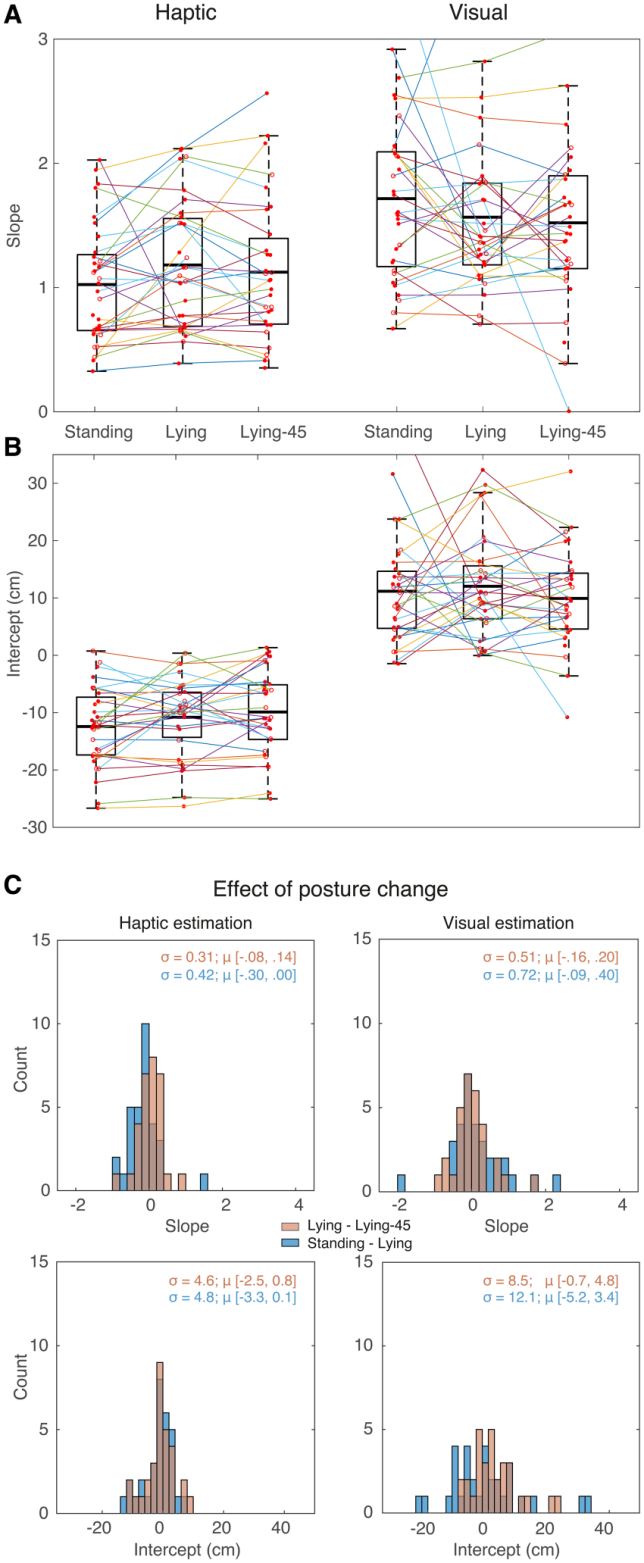
How the slopes and intercepts of the estimation tasks depend on posture. (A, B) Individual values for the slope (A) and the intercept (B). Symbols (connected by lines) indicate individual participants. The open symbols indicate participants whose results in the matching task (Figure 5) are unreliable. The boxes indicate the second and third quartiles; the thick horizontal line indicates the fixed effect of the group. The whiskers indicate the range of data, not considering outliers. (C) The distribution of the effect of posture change on the slope and the intercept. Selected bin widths were 0.2 for the slope and 2 cm for the intercept. For each distribution, we provide the standard deviation (σ) together with the 95% confidence interval for the mean (μ).

Participants were able to judge the different bar lengths: the estimated bar length scales linearly with the presented bar length (Figure 3). This figure shows the average normalised deviation for each bar length and participant, as well as the average across participants. The results in Figure 3 show two clear effects: the judgements differ considerably between participants and the visual judgements of length were larger than the haptic judgements. The latter result might correspond to an overestimation of the distance of the visual bar. To provide some more insight into the variability between participants, we analysed the individual regression parameters (Figure 4).

The individual values for the regression parameters (Figure 4A and 4B) show the same pattern as the raw data. Firstly, there was a clear mismatch between the visual and haptic estimates: the visual judgements of length were on average 20 cm larger than the haptic ones (difference in intercept). Correspondingly, the slopes tend to be larger for the visual estimates. Secondly, the judgements differ considerably between participants. This can be quantified by the overall standard deviation of the slopes and intercepts (averaged across the three postures): it is 0.52 and 6.9 cm for the haptic judgements and 0.68 and 8.9 cm for the visual judgements. The coefficient of variation was 0.48 and 0.43 for the haptic and visual slopes, and 0.63 and 0.81 for the haptic and visual intercepts. Moreover, Figure 4A shows that for most participants these parameters varied considerably between the postures (lines connecting individual participants’ data are not horizontal). There was no overall effect: for all the posture changes, the 95% confidence interval around the mean of the distributions of these effects includes zero (Figure 4C). The effects of posture on estimation were idiosyncratic: some participants show larger values when standing than when lying, whereas for others the difference is in the opposite direction. This idiosyncrasy could mean that individual participants’ judgements respond differently to the change of posture, or that the differences in judgements were related to other factors than the change in posture. We think it is unlikely that this idiosyncrasy would just be measurement noise, as some effects are reproducible. For instance, in Figure 3B, the largest visual estimates in the standing posture are across all values of Δl from the same participant (orange symbols). Another participant (yellow symbols) consistently produced the largest estimates in the lying-45 posture.

If the differences in Figure 4A and 4B are related to changes in postures, we would expect larger differences if the posture changes involved body parts that might be relevant for the size judgement. The posture of the arm is irrelevant for the visual size judgements, so these should differ less between the two lying postures than between a standing and a lying posture. Indeed, there seems to be a tendency for participants to be slightly more consistent in their lying postures compared to standing postures, as evidenced by connecting lines being closer to horizontal for both slopes and intercepts. This observation holds for both the slopes and intercepts. This tendency is also visible in Figure 4C, in which the distribution of the changes between postures is plotted. Again, we see for the visual judgements more consistency (a smaller distribution) from lying to lying-45 posture (orange histogram) than from standing to lying (blue histogram). However, Levene's test for equality of variances showed that these distributions did not differ significantly, for both comparisons: *F*(1, 63) < 3.0 and *p* > .08. So, larger posture differences do not lead to consistently larger changes in judgement. This makes it unlikely that individual differences in posture is the cause of the large idiosyncratic posture-to-posture judgement changes. Figure 4C furthermore shows that the haptic judgements were more consistent across postures than the visual judgements.

**Figure 5.**
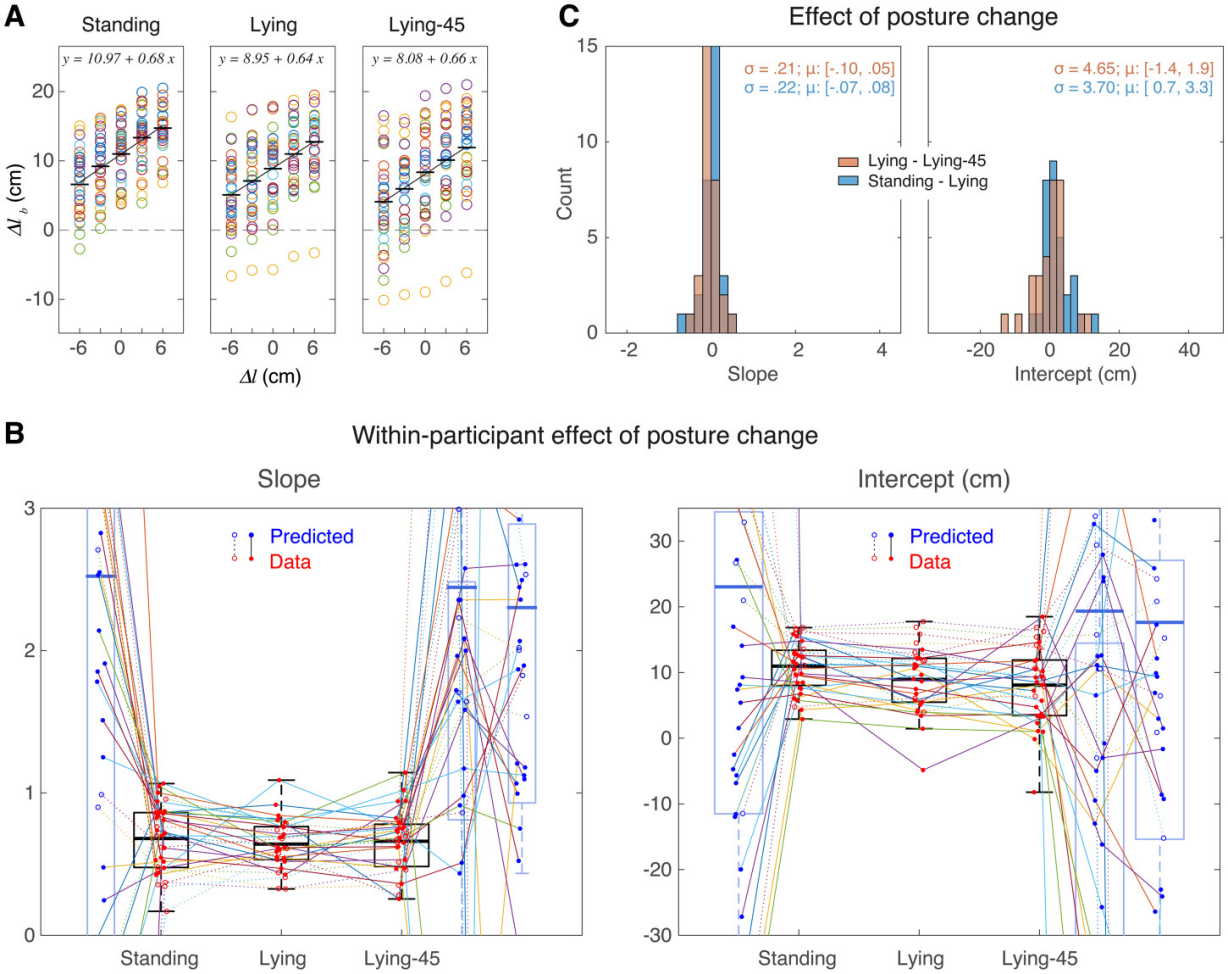
The data for the matching task. A expressed as the matched deviation $\Delta l_{b}$ . See Figure 3 for further details. (B, C) How the slopes and intercepts of the matching task depend on posture. See Figure 4 for further details. For comparison, we added in panel B the predictions for the three postures based on equation (11) as blue dots (connected to the red dots for the actual data) for each participant. Note that some predictions were very far off, resulting in one fixed effect for the predicted intercept lying-45 to fall outside the interquartile range of the individual predictions. Therefore, we decided not to plot all predictions. Open symbols and dotted connecting lines indicate that participants’ results are unreliable (see the section ‘Data Analysis’). (The colour versions available online)

Our second way to assess perceived size is the matching task. As in the estimation tasks, the relationship between the set and the presented length is linear for the presented range (Figure 5A). The overall standard deviation—averaged across the three postures—of the slopes and intercepts is 0.21 and 4.9 cm, respectively, corresponding to a coefficient of variation of 0.32 and 0.53. The variability between participants in matching is thus more than 25% smaller than in the estimation tasks. If the matching task would be based on matching the visual and haptic estimates we obtained in Figure 3, one would expect that the offset in visuo-haptic matching would correspond to the difference between the haptic and visual offsets, so more than 20 cm. However, we find an average offset of less than 10 cm.

We used equation (11) to make individual predictions for slope and intercept in the matching task (represented by the blue dots in Figure 5B) and plotted these together with the measured slopes and intercept (red dots in Figure 5B). On average, both the measured slopes and the intercepts are closer to zero than what we predicted on the basis of the results of the estimation task; so the mismatch between vision and haptics is smaller in matching than in estimation. Most importantly, the performance in size matching seems not very well correlated with the predictions that we made based on the individual size judgements.

## Discussion

We showed that participants show considerable biases in judging visual and haptic length but can nevertheless reliably judge small variations in these lengths (Figure 3). Although these judgements were on average not affected by changes in the orientation of the body or arms, individual participants’ judgements varied considerably between orientations, but these variations are presumably not due to the specifics of the posture. These results hold for both our methods to assess the visual and haptic length (magnitude estimation and matching).

The first finding to discuss is the large mismatch between visual and haptic length judgements: irrespective of the posture, we find that haptic lengths are underestimated both in estimation (Figures 3 and 4B) as in matching (Figure 5A and 5B). These mismatches have the opposite sign as the one reported by Kim et al. (2022). This difference might be related to our use of a VR headset to present the visual stimuli. In this way, we were able to keep the visual information constant across postures. However, this choice has a severe limitation, as we did not calibrate the headset for individual positioning of the participants’ eyes (and thus the inter-ocular distance), which will cause idiosyncratic errors (Renner et al., 2015).

VR headsets might also introduce systematic errors. The lack of calibration could cause this if the standard settings of the headset (e.g., interocular distance) deviate systematically from the characteristics of our sample. Furthermore, the accommodation distance is independent of the simulated distance, leading to the so-called accommodation–vergence conflict (Vienne et al., 2020). Indeed, it has been reported that egocentric distances of objects in VR are generally underestimated (Korshunova-Fucci et al., 2023; Renner et al., 2013). Can such underestimation explain the observed mismatch between visual and haptic perceived size? An underestimation of egocentric distances would correspond to an underestimation of its distance, and thus of its visual size. This would be in line with the results of Kim et al. (2022). In contrast, we found a systematic overestimation of length for the visual bar compared to the haptic bar, in terms of both slope as well as intercept. This result seems thus in conflict with the generally observed misjudgement of visual distances. However, we cannot make any strong claim, as we cannot assess whether the bias that we found is due to a visual misjudgement, as we assume in the above reasoning. Alternatively, the bias could be due to a haptic underestimation, as we do not know the bias in our participants’ estimate of the length of 1 m and therefore used normalised judgements.

If this lack of calibration is due to a fixed overestimation of visual distance, it would introduce a change in the scaling factor $s_{v}$ in equation (2), an effect our analysis method can deal with. However, if the misjudgement of visual distance depends on the retinal size (Sousa et al., 2011, 2012), it would result in a fitted line that would not pass through the origin, so the proportional relations we assume in equation (2) wouldn’t capture the behaviour. A similar limitation might hold for the haptic length: we assume a proportional relation in equation (1), but this might be incorrect, as the haptic length perception has no direct sensory input but is derived from the difference in position between the two hands. A small bias in the judged location of one hand would already result in a deviation from the proportional relation.

Based on the equations (5) and (6), we can make predictions for the relationship between slopes and/or intercepts. If the fitted values do not follow these predictions, our assumption of a proportional relationship is likely wrong. The first prediction is that the sum of the slopes is 2.0. The sum of the fitted values of the visual and haptic slopes are clearly larger (vary between 2.6 and 2.8 for all three postures; Figure 3). The second prediction is that the difference in intercepts of our linear fits ( $H_{0}\;$ and $V_{0}$ ) would be fully determined by the difference in slopes:

$$H_{0}-V_{0}=(V-H)l_{0}$$

As the difference between the slopes for the three postures is 0.7, 0.4, and 0.4 (Figure 3), and the shoulder width $l_{0}$ is about 40 cm, one would expect the difference between the intercepts to be 28, 16 and 16 cm, which differ considerably from the 23.6, 22.8, and 19.8 we found (Figure 3). This limitation of our theoretical approach prevents us from drawing strong conclusions about the nature of the biases but does not influence our main conclusion that participants show considerable biases in judging visual and haptic length but can nevertheless reliably judge small variations in these lengths.

A remarkable finding is that the matching lengths are inconsistent with the predictions one would make based on the judged lengths: the average biases in the matching task are smaller than one would predict (Figure 5B). This inconsistency resembles inconsistencies that have been reported before between related matching tasks of position (Kuling et al., 2017) or size (Figure 2 of Kopiske & Domini, 2018). These inconsistencies presumably arise because matching is not based on matching sensory representations but on a transformation of sensory information from one modality to the other. If biases are introduced in these transformations, rather than in internal representations, it is not surprising that the biases differ between the tasks. Assuming that the implementation of this transformation varies between participants, this line of reasoning might also explain the idiosyncrasy of the biases. This mismatch between vision and haptics might be partially due to a ceiling effect: the participants that reached the limits of the bar indeed had shallower slopes and larger intercepts (open symbols connected by dotted lines in Figure 5B. If we had excluded these participants, we would have underestimated the visuo-haptic mismatch.

An interesting finding is that the visual judgements are not more consistent than the haptic ones, both within participants (standard deviation of an individual) and across participants (the variability seen in Figure 4). Although this conflicts with the widely believed claim that vision dominates (Rock & Harris, 1967), it is in line with more recent research (Ernst et al., 2000; van Beers et al., 2011), and can be explained in terms of the transformation we discussed in the previous paragraph. The reason that vision frequently appears to be more reliable is that experiments generally involve a task that is defined in visual rather than haptic space. Therefore, visual information can directly be used, whereas haptic information requires a transformation to the visual task coordinates. A second reason for the low reproducibility of visual judgements might be the use of VR with the head fixed. This has decoupled our participants from the world, so they could not recalibrate vision, for instance, using one's body height or surrounding familiar objects. In contrast, by touching one's own body between trials, participants received feedback in between trials, which allowed haptic position sense to continuously calibrate.

We introduced the lying-45 condition because it loads the flexors of the elbow, and loading muscles has been reported to influence joint position sense (Smith et al., 2009; Supralk et al., 2007). We already mentioned in the introduction that not all studies found such an effect. In line with the report of Kuling et al. (2013) that loading did not affect haptic judgements of hand position of a single arm, we also did not find an effect of the manipulation of elbow load on size perception using both arms.

Note that the variability in the effect of postures on slope and intercept (Figure 4C) is not much smaller than the variability in the slope and intercept themselves; the ratio ranges from 0.32 (matching slope) to 0.93 (visual intercept). These considerable changes in the bias across postures seemed unrelated to the specifics of the postures, as the changes were comparable for a change of the orientation of the whole body (standing vs. lying) as for the change of orientation of only the lower arm (lying vs. lying-45). A possible explanation for this is that whenever our participants were brought in a new posture, they had to estimate the relevant transformation suitable for that posture. Every new estimation introduces a new random error in the transformation. If a new estimate for the transformation is made at the beginning of each block and remains fixed within that block, this process might explain why there is such a large variability between blocks (and thus postures), despite the consistency within a block.

A last finding to discuss is that the results of the matching task were more than 25% less variable than the estimation task. A simple explanation is that the bias for the estimation task is based on estimations in different trials that were even in different blocks, whereas, for the matching task, both estimates were formed and combined in a single trial. It is therefore very likely that several sources of variability won’t affect the matching outcome, but will affect the bias based on estimations.

We started this research to answer whether the effect of body orientation on visuo-haptic size matching (Kim et al., 2022) was due to effects on the visual judgements or due to an effect on the haptic judgements. Unfortunately, our results failed to answer this question at three levels. The first level is that although our paradigm produced a systematic visuo-haptic mismatch, the sign of the mismatch was opposite to that of the original study. The second level at which we failed is that we did not find a systematic effect of body orientation on the mismatch. Lastly, we found that the mismatch based on directly matching a visual and haptic percept was considerably smaller than the mismatch we derived for visual and haptic size estimates. In summary, we conclude conclusions about perceived sizes of objects are very sensitive to details of the experimental approach.
